# Conserved Transcriptional Responses to Nutrient Stress in Bloom-Forming Algae

**DOI:** 10.3389/fmicb.2017.01279

**Published:** 2017-07-18

**Authors:** Matthew J. Harke, Andrew R. Juhl, Sheean T. Haley, Harriet Alexander, Sonya T. Dyhrman

**Affiliations:** ^1^Lamont-Doherty Earth Observatory, Columbia University Palisades, NY, United States; ^2^Department of Earth and Environmental Sciences, Columbia University Palisades, NY, United States; ^3^Department of Population Health and Reproduction, University of California, Davis Davis, CA, United States

**Keywords:** transcriptome, MMETSP, nitrogen, phosphorus, conserved response, phytoplankton

## Abstract

The concentration and composition of bioavailable nitrogen (N) and phosphorus (P) in the upper ocean shape eukaryotic phytoplankton communities and influence their physiological responses. Phytoplankton are known to exhibit similar physiological responses to limiting N and P conditions such as decreased growth rates, chlorosis, and increased assimilation of N and P. Are these responses similar at the molecular level across multiple species? To interrogate this question, five species from biogeochemically important, bloom-forming taxa (Bacillariophyta, Dinophyta, and Haptophyta) were grown under similar low N, low P, and replete nutrient conditions to identify transcriptional patterns and associated changes in biochemical pools related to N and P stress. Metabolic profiles, revealed through the transcriptomes of these taxa, clustered together based on species rather than nutrient stressor, suggesting that the global metabolic response to nutrient stresses was largely, but not exclusively, species-specific. Nutrient stress led to few transcriptional changes in the two dinoflagellates, consistent with other research. An orthologous group analysis examined functionally conserved (i.e., similarly changed) responses to nutrient stress and therefore focused on the diatom and haptophytes. Most conserved ortholog changes were specific to a single nutrient treatment, but a small number of orthologs were similarly changed under both N and P stress in 2 or more species. Many of these orthologs were related to photosynthesis and may represent generalized stress responses. A greater number of orthologs were conserved across more than one species under low P compared to low N. Screening the conserved orthologs for functions related to N and P metabolism revealed increased relative abundance of orthologs for nitrate, nitrite, ammonium, and amino acid transporters under N stress, and increased relative abundance of orthologs related to acquisition of inorganic and organic P substrates under P stress. Although the global transcriptional responses were dominated by species-specific changes, the analysis of conserved responses revealed functional similarities in resource acquisition pathways among different phytoplankton taxa. This overlap in nutrient stress responses observed among species may be useful for tracking the physiological ecology of phytoplankton field populations.

## Introduction

Phytoplankton productivity is frequently limited by the supply of phosphorus (P) and nitrogen (N) (Tyrrell, 1999; Elser et al., 2007). Although the concentration and composition of these nutrients is well known to shape phytoplankton communities and influence physiological responses, the diversity of metabolic responses that underpin phenotypic changes in phytoplankton remains unknown in many cases. Gaining insight into these metabolic responses that drive phenotypic change can help define the physiological plasticity of organisms and their concomitant influence on marine food webs and carbon export (Azam et al., 1983; Longhurst and Glen Harrison, 1989; Ducklow et al., 2001; DeBiasse and Kelly, 2016).

Transcriptome profiling allows for the study of the metabolic and physiological response of organisms to stressors (Hwang et al., 2008; Thompson et al., 2011; Dyhrman et al., 2012), and comparing transcriptomic responses across species can illuminate conserved and divergent traits. For instance, Bender et al. (2014) observed similar physiological responses to N stress in three phylogenetically and physiologically distinct species of diatom (two raphid and one centric). These included a reduction in carbon fixation as well as carbohydrate and fatty acid metabolism, with an increase in N recycling (Bender et al., 2014). A comparison of four prymnesiophytes found a set of core genes shared among all species mapping to essential metabolic pathways such as the biosynthesis of amino acids, carbon metabolism, fatty acid metabolism, and purine and pyrimidine metabolism (Koid et al., 2014). In another example, co-occurring flagellates were found to have similar urea transporters but diverse urease genes and N uptake and assimilation pathways, suggesting varying strategies of survival in the N-limited Arctic Ocean (Terrado et al., 2015). However, few studies have investigated the response similarities and differences of more divergent taxa grown under the same laboratory conditions and sequenced in a similar manner.

The three phyla studied herein, Bacillariophyta, Dinophyta, and Haptophyta, represent some of the most abundant and globally distributed eukaryotic phytoplankton groups present in the ocean (Sournia et al., 1991; de Vargas et al., 2015) with important roles in global biogeochemical cycles (Milliman, 1993; Jordan and Chamberlain, 1997; Bopp et al., 2005). Diatoms are thought to contribute 40% of marine primary productivity (Falkowski et al., 2004) and up to 20% of global carbon fixation (Matsuda, 2014), and calcifying haptophytes can have significant impacts on ocean pH and alkalinity through photosynthesis and calcification (Paasche, 2001). The diversity and importance of dinoflagellates continues to be explored, filling niches as autotrophs, mixotrophs, and grazers as well as existing in complex associations with other organisms (Hackett et al., 2004; Taylor et al., 2007; Stern et al., 2010). Blooms of species from each of these phyla can have dramatic impacts on coastal and open ocean ecosystems, fisheries, and recreation (Johansson and Granéli, 1999; Smayda, 2002; Glibert et al., 2005; Heil et al., 2005).

Phytoplankton are known to exhibit similar physiological responses to limiting N and P conditions such as decreased growth rates, chlorosis, and increased assimilation of N and P. Are these responses similar at the molecular level across taxonomic groups? In the current study, we sought to interrogate this fundamental question by examining the transcriptional profiles of five bloom-forming species (toxic and non-toxic, calcifying and non-calcifying) exposed to limiting N and P conditions. As part of the Marine Microbial Eukaryotic Transcriptome Project (MMETSP; Keeling et al., 2014), five species of phytoplankton representing important bloom-forming species from three globally important phyla (Bacillariophyta, Dinophyta, and Haptophyta) were grown under low N, low P, and replete nutrient conditions. The species were chosen because they form blooms but have distinct traits even within a grouping, varying in their ability to produce toxins or calcify, among other factors. The transcriptomes were compared and examined for conserved molecular responses in transcription and concomitant shifts in physiology.

## Materials and methods

### Culture conditions

Five species of phytoplankton representing important bloom-forming species from three globally important phyla (Bacillariophyta, Dinophyta, and Haptophyta) were examined in this study. These species included the toxic, bloom-forming dinoflagellate, *Alexandrium monilatum* CCMP3105 (isolated from the Gulf of Mexico, Gulfport, Mississippi, USA, 2000), the bloom-forming dinoflagellate, *Prorocentrum minimum* CCMP1329 (isolated from Great South Bay, NY, USA, 1958), the cosmopolitan diatom, *Chaetoceros affinis* CCMP159 (isolated from the Gulf of Mexico, May 1980), and the haptophytes *Chrysochromulina polylepis* CCMP1757 (isolated from the North Sea, 1988) and *Gephyrocapsa oceanica* RCC1303 (isolated from Arachon Bay, France, Jan 1999).

Cultures of all species were grown in modified L1 medium to control for nitrate and phosphate concentration in each treatment (replete, low N, and low P). Initial ratios of N:P added to the growth media were modified for all species as follows: replete = 16:1 (576 μM NO^3−^: 36 μM ${\text{PO}}_{4}^{3-}$), low N = 0.1:1 (5 μM NO^3−^: 36 μM ${\text{PO}}_{4}^{3-}$), and low P = 5760:1 (576 μM NO^3−^: 0.1 μM ${\text{PO}}_{4}^{3-}$) except for *G. oceanica* which was grown at the following N:P ratios: replete = 17:1 (100 μM NO^3−^: 6 μM ${\text{PO}}_{4}^{3-}$), low *N* = 0.2:1 (1 μM NO^3−^: 6 μM ${\text{PO}}_{4}^{3-}$), and low P had no phosphate added (100 μM NO^3−^: 0 μM ${\text{PO}}_{4}^{3-}$). The lower added nutrient concentrations for *G. oceanica* were necessary to ensure consistent calcification. Cultures were grown with a light intensity of 100 μmol quanta m^−2^ s^−1^ on a 14:10 light:dark cycle and temperature varied by species (according to species-specific temperature preferences) with *A. monilatum* grown at 24°C, *C. affinis, G. oceanica*, and *P. minimum* grown at 18°C, and *C. polylepis* grown at 15°C. Some cultures were continuously rotated on an orbital shaker (*P. minimum*, 75 rpm; *C. affinis*, 100 rpm; *C. polylepis*, 100 rpm) to maintain optimal growth. Although cultures were not axenic, they were uni-algal and uni-eukaryotic.

### Experimental design

**Figure 1:**
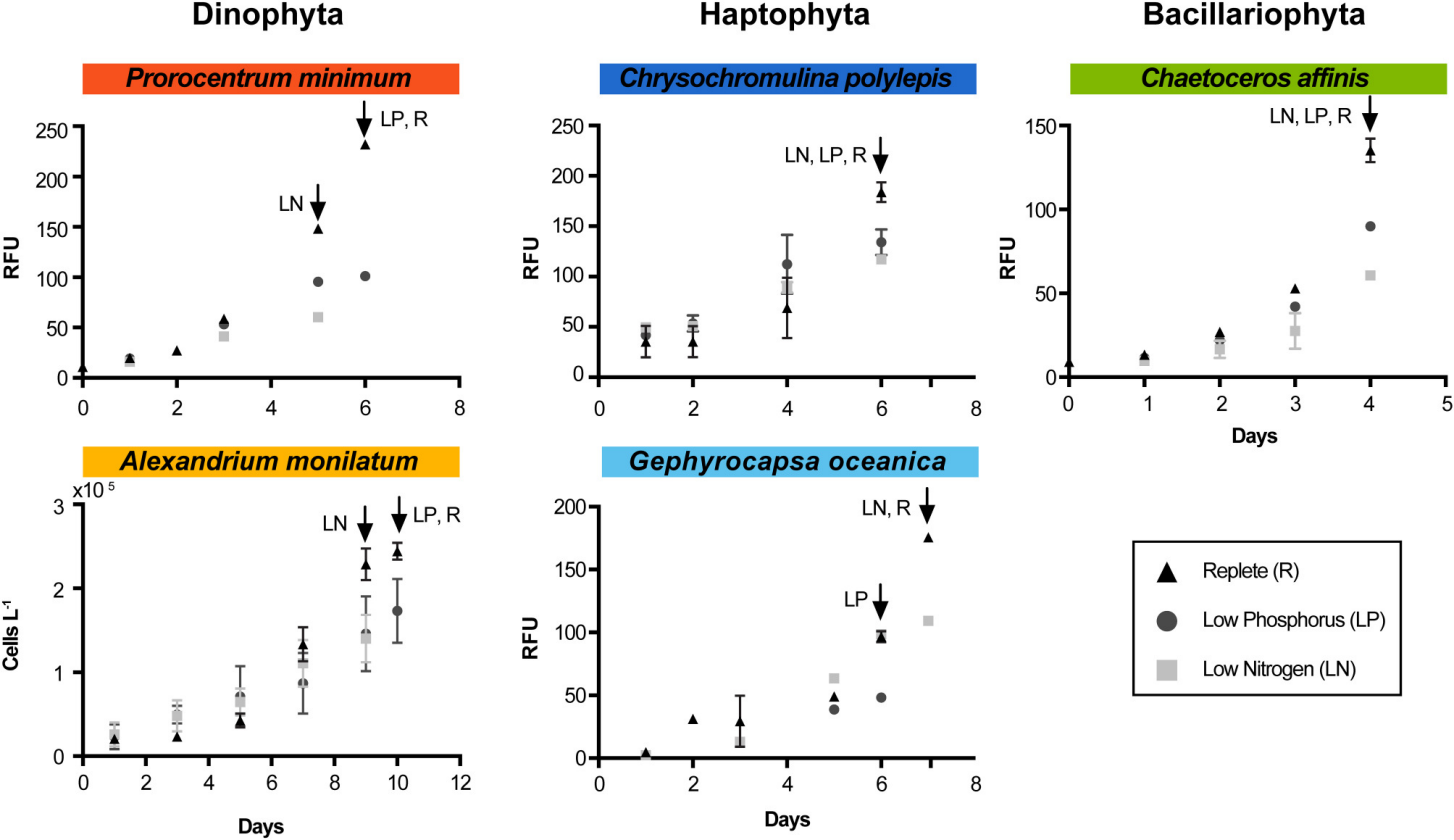
Growth curves for each species and each treatment during the experiment. Arrows indicate time of harvest for each treatment (designated by treatment abbreviation). RFU indicates “relative fluorescence unit.” Triangles represent the replete condition (control), squares represent the low N, and circles represent the low P. Error bars are the standard error of the mean (*n* = 3). For *A. monilatum*, growth curves were determined by cell counts due to difficulty in measuring reliable *in vivo* fluorescence.

For all species, single entrainment cultures (100 mL) were grown on modified L1 medium (replete, low N, and low P, as described above) for 3 days. At the start of the experiment, triplicate 2-L bottles (1 L experimental volume) were inoculated with 25 mL of corresponding entrainment culture (replete, low N, and low P) into the same modified L1 medium. The entrainment step was used to minimize nutrient carry over. The experimental cultures were monitored at the same time daily for *in vivo* fluorescence, cell counts, and photosynthetic efficiency. Additional samples were obtained at the time of harvest for particulate organic carbon (POC), particulate organic nitrogen (PON), total chlorophyll *a*, and particulate carbohydrates (pCHO). Replete cultures were harvested during exponential phase growth and the low N and low P treatments were harvested when growth rates decreased relative to the replete (Figure 1). This sampling scheme allowed for the identification of N and P effects independently, and a similar approach has been used for previous gene expression studies (Dyhrman et al., 2006a; Bender et al., 2014; Haley et al., in press). Nutrient stress, here defined as a physiological state whereby the availability of a limiting nutrient decreases relative to cellular demand, causing a decline in rates of photosynthesis and growth, will be used to describe the N- and P-limited culture states throughout the manuscript (MacIntyre and Cullen, 2005).

### Culture analysis and biochemical measurements

Samples for determination of cell concentration by microscopy were preserved in acid Lugol's solution (2% final concentration). Maximum quantum efficiency of photosystem II (F_v_/F_m_) was estimated from *in vivo* (F_0_) and DCMU (3,4-dichlorophenyl-1,1-dimethylurea)-enhanced *in vivo* fluorescence (F_m_) of each replicate experimental sample on a Turner Designs TD-700 fluorometer. Chlorophyll *a* was measured by filtering 10 ml of culture onto a Whatman GF/F (25 mm). Filters were then stored at −20°C prior to extraction. Chlorophyll *a* was extracted in 10 ml of 90% acetone, vortexed for 15 s at maximum speed, and stored in the dark at −20°C for 12 h. After extraction, each sample was centrifuged and chlorophyll *a* concentration was determined from fluorescence of the supernatant before and after acidification (Strickland and Parsons, 1972) using a Turner Designs Aquafluor fluorometer. The fluorometer was calibrated using chlorophyll *a* from *Anacystis nidulans* (Sigma). For pCHO measurements, 25 ml of culture was filtered onto pre-combusted Whatman GF/F (25 mm), and filters were then stored at −20°C prior to their analysis by a modified phenol-sulfuric acid method (Dubois et al., 1956). Briefly, filters were soaked in 5% phenol and concentrated sulfuric acid in the original sample tubes at 30°C for 20 min. The filter was then removed, and the light absorption of the sample at 490 nm was measured using a Hach DR2700 spectrophotometer. Sample absorbance was compared to a glucose standard curve and pCHO values are expressed as pmoles of glucose equivalents per cell. Samples to determine POC/PON (50 ml) were filtered through pre-combusted (450°C for 5 h) Whatman GF/F filters (25 mm) and stored in pre-combusted foil packets at −20°C. POC/PON was measured at the Nutrient Analytical Services Laboratory at the Chesapeake Bay Laboratory (University of Maryland, Solomons, MD) on a CE-440 Elemental Analyzer following the USEPA protocol (Zimmermann et al., 1997) for POC/PON.

Homogeneity of variance was confirmed for the results of the measurements listed above by the Brown-Forsythe test. Then, one-way ANOVAs followed by Fisher's least significant difference (LSD) *post hoc* tests were used to determine whether the means of low N and low P were significantly different (α = 0.05) from means of the replete cultures.

A series of follow-up experiments using the methods and timing outlined above, were conducted to assay alkaline phosphatase activity (APA). For APA analysis, triplicate biological samples (25 mL) from the repeated replete and low P experiments were filtered onto 47-mm polycarbonate membranes (0.2 μm) and stored at −20°C until analysis. APA was assayed after Dyhrman and Ruttenberg (2006) using the fluorogenic phosphatase substrate 6,8-difluoro-4-methylumbelliferyl phosphate and normalized to cell count. Cell-specific APA in the replete and low P treatments were compared using an unpaired *t*-test.

### RNA extraction and sequencing

At the time of harvest, triplicate 150-mL subsamples of each replicate in each treatment and species were filtered onto 47-mm polycarbonate filters (*A. monilatum*: 8 μm, *P. miminum*: 5 μm, *C. affinis*: 3 μm, *G. oceanica*, and *C. polylepis*: 1 μm). The filters were immediately flash frozen and stored in liquid nitrogen. Total RNA was extracted with the RNeasy Mini Kit (Qiagen, Valencia, CA) with a modified lysis procedure. Lysis was conducted by adding 1.4 mL Buffer RLT and ~250 μl zirconium/silica beads (0.5 mm), and samples were then vortexed for 1 min at 250 rpm. Total RNA was then treated with TurboDNase (Ambion, Austin, TX) according to the manufacturer's protocol to remove remaining genomic DNA. RNA from the three replicate cultures from each experimental condition was pooled prior to sequencing to average across biological variability in transcriptional response between replicate flasks. Total RNA was quantified using a Qubit fluorometer (Invitrogen, Carlsbad, CA) and quality was assessed using the Agilent 2100 Bioanalyzer (Agilent, Santa Clara, CA). Libraries were generated at the National Center for Genome Resources (NCGR, Santa Fe, NM) using Illumina TruSeq RNA Sample Preparation Kit from ~2 μg of total RNA. Sequencing of 50 base pairs (bp) paired-end reads from each library was performed on an Illumina HiSeq 2000 at the NCGR. Sequence data is available on *i*Microbe (http://data.imicrobe.us/project/view/104) under the following identification numbers *A. monilatum*: MMETSP0093, MMETSP0095, and MMETSP0096; *P. minimum*: MMETSP0053, MMETSP0055, and MMETSP0056; for *C. affinis*: MMETSP0088, MMETSP0090, and MMETSP0091; *C. polylepis*: MMETSP0143, MMETSP0145, and MMETSP0146; *G. oceanica*: MMETSP1363, MMETSP1364, and MMETSP1365.

### Transcriptome assembly, annotation, and read counts

As part of the MMETSP (Keeling et al., 2014; Caron et al., 2017), a combined transcriptome assembly of all treatments for each species was carried out using NCGR's internal pipeline (Batch Parallel Assembly version 2.0). Sequence reads from all treatments were preprocessed using the SGA preprocess (Simpson and Durbin, 2012) for quality trimming (swinging average) at Q15. Reads less than 25 bp after trimming were discarded. Preprocessed sequence reads from each individual sample were assembled into contigs with ABySS v. 1.3.3 (Simpson et al., 2009), using 20 unique k-mers between *K* = 26 and *K* = 50. ABySS was run requiring a minimum k-mer coverage of 5, popping at > 0.9 branch identity, with the scaffolding flag disabled to avoid over reduction of divergent regions. Unitigs from all k-mer assemblies and all samples were combined and redundancies were removed using CD-HIT-EST (Li and Godzik, 2006) with a clustering threshold of 98% identity. The overlap layout consensus (OLC) assembler CAP3 (Huang, 1999) was used to identify minimum 100 bp overlaps between the resultant contigs and assemble larger sequences. The resulting contigs were paired-end scaffolded using ABySS (Simpson et al., 2009). Sequence read pairing information was used in GapCloser (v. 1.10) from SOAP *de novo* assembly software (Li et al., 2008) to walk in on gaps created during scaffolding. Redundant sequences were again removed using CD-HIT-EST at a clustering threshold of 98% identity. The consensus contigs were filtered at a minimum length of 150 bp to remove probable incomplete contigs, producing the final set of contigs. Read counts were generated using RSEM (Li and Dewey, 2011) with default parameters to align sequence reads back to the combined assembly contigs and predicted coding sequences. The relative completeness of the assembly was assessed using the core eukaryotic gene-mapping approach (CEGMA; Parra et al., 2007).

Initial annotations were derived through the MMETSP annotation bundle, where coding sequences were predicted using ESTScan (Iseli et al., 1999; Lottaz et al., 2003) with a Bacillariophyta scoring matrix. Sequence reads were aligned back to the nucleotide motifs of the predicted coding sequences using BWA (Li and Durbin, 2009). Peptide predictions over 30 amino acids in length were annotated. Matches to the UniProtKB/Swiss-Prot database were identified via blastp (Altschul et al., 1990) searches. Protein sequences were also functionally characterized using HMMER3 (Zhang and Wood, 2003) against Pfam-A (Finn et al., 2010), TIGRFAM (Haft, 2001), and SUPERFAMILY (Gough et al., 2001) databases. KEGG (Kyoto Encyclopedia of Genes and Genomes) biochemical pathways for each contig were identified with KEGG Automatic Annotation Server (KAAS) using the partial genome single-directional best-hit method (Kanehisa et al., 2006; Moriya et al., 2007). The protein sequences were blastp searched against the uniref90 database (Suzek et al., 2015) with Diamond (Buchfink et al., 2015). All annotations discussed in the text should be considered putative. PCA and confidence ellipses of the proportion of reads mapped to KEGG ID's by species were calculated and visualized with the FactorMineR package in R. Heat maps of these data were visualized with Morpheus (https://software.broadinstitute.org/morpheus/).

### Ortholog identification

To identify orthologous groups shared between species, OrthoMCL v2.0.9 was used to filter peptides on length and maximum number of stop codons (10 and 20, respectively). Filtered peptides from each species were then merged and an all-vs.-all blast was conducted using Diamond (Buchfink et al., 2015). The resulting blast output file was clustered with MCL (Enright, 2002) using the inflation parameter of 1.4. Orthologs were annotated at the contig-level by building consensus annotation from all available data.

### Differential expression analysis and statistics

Significant differential abundance patterns in the low N and low P treatments vs. the replete condition were assigned using an empirical Bayes method for single replicate experiments (ASC; Wu et al., 2010). ASC estimates the prior distribution by modeling biological variability using the data itself, rather than imposing a negative binomial distribution. Contigs with a fold change greater than or equal to 2 and a posterior probability (post-*p*) greater than 0.95 were deemed significantly differentially abundant, which are parameters used in previous studies of this type (Dyhrman et al., 2012; Konotchick et al., 2013; Frischkorn et al., 2014; Kopf et al., 2015). For analysis of orthologs, non-normalized read counts assigned to contigs falling within an ortholog for each species and treatment were summed to generate an ortholog read count for each species and treatment. These data were then input into ASC as above to investigate the relative abundance of orthologs in treatments compared to the control at the species level. Results for both analyses can be found in Tables S1, S2.

To investigate gene set (contigs grouped by KEGG pathway) enrichment patterns (Subramanian et al., 2005), the transcripts per million (TPM) for each contig in each treatment were normalized to the average abundance of that contig across the three treatments (replete, low N and low P) after Haley et al. (in press). This resulted in three distributions of normalized enrichments of contigs within the gene set for each treatment. The normalization step was necessary to equalize the relative contribution of each contig to the gene set, thus avoiding bias whereby contigs within the gene set have different inherent abundance levels. The three distributions were then compared using Kolmogorov-Smirnov tests, with significance at *p* ≤ 0.05, to examine the null hypothesis that normalized gene set enrichments in the low N and low P treatments had the same distribution across contigs as the replete treatment. Hence, rejecting the null hypothesis for a treatment indicated a significantly different pattern of normalized contig abundance within the specified gene set compared to the replete treatment. Since the test does not specify how the distributions differ, when gene sets are described as significantly enriched (or significantly less abundant) relative to the replete, the description implies a significant test result, combined with a consistent direction of differential abundance across contigs within the gene set, a direction apparent in plots of the distributions. However, we note the caveat that the test is sensitive to consistent directionality across the gene set; if some contigs within a gene set were more enriched while at the same time other contigs within the gene set were less abundant, the test could fail to reject the null hypothesis even if the enrichments for the individual contigs within the gene set were very large. Cases where this is potentially occurring are noted where applicable. Kolmogorov-Smirnov tests were conducted with Prism (GraphPad Software, La Jolla, CA), which uses a bootstrapping approach to calculate exact *p*-values for 2-sample Kolmorogov-Smirnov tests. Significance was determined at *p* ≤ 0.05.

## Results and discussion

The extent to which resource availability shapes eukaryotic phytoplankton assemblages and how individual taxa respond to resource changes is a critical and ongoing area of study (Azam and Worden, 2004; de Vargas et al., 2015). Understanding how bloom-forming phytoplankton respond to alterations in N and P is vital to enhancing our predictive power as these species can have profound impacts on coastal and open ocean ecosystems and global carbon biogeochemistry. Here, transcriptome profiling was used to compare the shared (conserved) mechanisms that different phytoplankton groups use to respond to nutrient stress and how these mechanisms could be linked to changes in physiological activity and biochemical pools. Although each species has a unique gene complement and response, focusing on conserved responses provides data that may be generalized within and across taxonomic groups.

### Species-specific biochemical pools, metabolic response, and assembly analysis

Nutrient stress typically caused a significant decrease in growth rate and/or cell yield relative to the control (Figure 1, Table 1). This shift in growth was coincident with a number of phenotypic changes. In all species, ratios of C:N increased under both N and P stress by 15–51% from replete conditions, with significance detected in all species under N stress and for *A. monilatum, P. minimum*, and *G. oceanica* under P stress (*p* < 0.05; Table 1). Further, where measured, photosynthetic efficiency (Fv/Fm) declined under N and P stress, with significant reductions observed under N stress for *G. oceanica* and *C. affinis* (*p* < 0.05; Table 1) and under P stress for *P. minimum* (*p* < 0.05; Table 1). Particulate carbohydrates (pCHO) were higher in both nutrient-stressed treatments (low N and low P) relative to the replete treatment, except for *A. monilatum* under N stress (*p* < 0.05; Table 1). In some cases, phenotypic responses were observed in only one taxonomic group. For example, particulate organic carbon (POC) significantly increased for only the dinoflagellates under N and P stress, while these stressors elicited significant decreases in particulate organic nitrogen (PON) and chlorophyll *a* in the diatom (*p* < 0.05; Table 1). Although particulate organic P concentrations were not measured, parallel experiments conducted in the same way yielded significantly elevated APA in low P treatments for *C. affinis, G. oceanica*, and *P. minimum* (*p* < 0.05; Table S3), a common response to P stress (Dyhrman, 2016). Collectively, the phenotypic changes were broadly consistent with what has previously been observed in other diatoms, haptophytes, and dinoflagellates under N and P stress (Hecky and Kilham, 1988; Dyhrman, 2016; Raven and Giordano, 2016). For example, increased POC under N stress has been observed in the diatom *Scrippsiella trochoidea* and the dinoflagellates *A. fundyense* and *A. monilatum* (Juhl, 2005; Eberlein et al., 2016). Carbohydrates often increase under nutrient stress in phytoplankton (Utting, 1985; Harrison et al., 1990) and nutrient stress is known to cause increased production of organic matter (including pCHO) in the haptophyte *Emiliania huxleyi* (Borchard and Engel, 2012). Despite some variability in species-specific responses, the phytoplankton examined here clearly modulated their phenotypes in response to nutrient stress, with many similarities between species and to similar studies on related species.

**Table 1:** Biochemical measurements at the time of harvest.

***A. monilatum***	**Replete**	**Low N**	**Low P**
Growth rate (μ)	0.26 (10%)	0.20 (17%)	0.22 (26%)
POC (pmoles/cell)	781.7 (8%)	1328 (5%)^****^	1186 (6%)^***^
PON (pmoles/cell)	175.3 (4%)	187.3 (3%)	222.1 (10%)^*^
C:N	4.5 (5%)	7.1 (7%)^****^	5.4 (4%)^*^
pCHO (pmoles/cell)	402 (31%)	213 (20%)	856 (39%)^*^
Chl a (μg/cell)	4.9E-05 (67%)	8.5E-05 (11%)	5.9E-05 (74%)
Fv/Fm	N/A	N/A	N/A
***P. minimum***			
Growth rate (μ)	0.48 (5%)	0.33 (4%)^****^	0.34 (1%)^****^
POC (pmoles/cell)	34.2 (17%)	59.7 (14%)^**^	73.4 (4%)^***^
PON (pmoles/cell)	3.3 (21%)	2.5 (8%)	4.1 (6%)
C:N	11.9 (4%)	23.9 (18%)^***^	18.0 (2%)^*^
pCHO (pmoles/cell)	28.4 (55%)	43.7 (30%)	53.9 (1%)^*^
Chl a (μg/cell)	1.3E-06 (31%)	1.3E-06 (28%)	2.4E-06 (22%)^*^
Fv/Fm	0.59 (5%)	0.58 (2%)	0.55 (2%)^*^
***C. affinis***			
Growth rate (μ)	0.80 (6%)	0.57 (2%)^***^	0.62 (1%)^*^
POC (pmoles/cell)	14.1 (5%)	13.2 (12%)	10.0 (64%)
PON (pmoles/cell)	2.1 (6%)	0.96 (17%)^*^	0.97 (61%)^*^
C:N	6.8 (5%)	12.0 (26%)^*^	9.9 (12%)
pCHO (pmoles/cell)	5.6 (50%)	10.7 (6%)^*^	7.1 (2%)
Chl a (μg/cell)	3.1E-06 (11%)	5.3E-07 (8%)^****^	1.9E-06 (6%)^**^
Fv/Fm	0.66 (4%)	0.36 (5%)^****^	0.66 (1%)
***C. polylepis***			
Growth rate (μ)	0.33 (5%)	0.21 (3%)^***^	0.23 (7%)^***^
POC (pmoles/cell)	45.1 (64%)	52.5 (28%)	56.9 (49%)
PON (pmoles/cell)	5.5 (55%)	5.1 (26%)	6.3 (46%)
C:N	7.6 (21%)	10.2 (3%)^*^	8.9 (5%)
pCHO (pmoles/cell)	24.3 (21%)	28.1 (33%)	34.0 (65%)
Chl a (μg/cell)	6.5E-06 (78%)	6.8E-06 (23%)	9.0E-06 (48%)
Fv/Fm	0.46 (27%)	0.42 (14%)	0.44 (2%)
***G. oceanica***			
Growth rate (μ)	0.83 (14%)	0.55 (2%)^**^	0.45 (6%)^**^
POC (pmoles/cell)	0.64 (61%)	0.85 (1%)	1.01 (2%)
PON (pmoles/cell)	0.067 (61%)	0.069 (3%)	0.044 (4%)
C:N	9.6 (3%)	19.4 (4%) ^****^	14.6 (4%)^****^
pCHO (pmoles/cell)	0.17 (80%)	0.24 (4%)	0.37 (11%)^*^
Chl a (μg/cell)	1.02E-07 (71%)	5.95E-08 (10%)	8.07E-08 (13%)
Fv/Fm	0.67 (2%)	0.62 (3%)^*^	0.64 (2%)

Values represent the mean (n = 3) with coefficient of variation (CV), the ratio of standard deviation to the mean, in parentheses. Asterisks indicate significant differences from replete, where

* *p < 0.05*,

** *p < 0.005*,

*** p < 0.0005, and

**** *p < 0.0001 (one-way ANOVA with Fisher's LSD post-hoc test). Elemental ratios are molar. N/A, not measured*.

The diatom *C. affinis* had the smallest transcriptome assembly, averaging 15.3 Mbp across the three treatments, which generated 19,072 contigs (Table 2). By comparison, the genome of the diatom *Thalassiosira pseudonana* generated 11,242 protein-coding genes (Armbrust et al., 2004) while the genome for *Phaeodactylum tricornutum* predicted 10,402 protein-coding genes (Bowler et al., 2008). The largest transcriptomes were observed in the dinoflagellates (*A. monilatum, P. minimum*), averaging 65.3 Mbp across the three treatments, with >96,000 contigs (Table 2), a finding consistent with the presence of large gene families and large genome sizes in dinoflagellates (1.5–112 Gbp; Murray et al., 2016) and with prior studies (Bayer et al., 2012; Beauchemin et al., 2012; Cooper et al., 2016). The transcriptome size for the two haptophytes fell between the diatom and dinoflagellates with *G. oceanica* producing 38,577 contigs from an average of 28.6 Mbp across the three treatments and *C. polylepis* producing 53,786 contigs from an average of 32.5 Mbp across the three treatments (Table 2). Assessments of assembly completeness using the CEGMA pipeline indicate the transcriptomes captured between 77 and 89% of the core eukaryotic genes, which is consistent with transcriptome studies in other marine algae (Di Dato et al., 2015; Meyer et al., 2015; Cooper et al., 2016). Efforts at annotation resulted in 23–57% of the transcriptomes annotated through the NCGR pipeline or blastp assignments against the uniref90 database, while KEGG orthology assignments were found for 14–27% of the transcriptomes (Table 2). These results mirror prior transcriptomic studies of eukaryotes (Koid et al., 2014; Cooper et al., 2016) and reflect the continued difficulty in annotating the transcriptomes of non-model organisms (Caron et al., 2017).

**Table 2:** Sequencing, assembly, annotation, and CEGMA completeness results for each species.

**Species**	**Transcriptome size [Mbp]**			**Combined Assembly**	**Percent annotated**			**CEGMA**
	**Replete**	**Low N**	**Low P**	**# Contigs**	**NCGR (%)**	**uniref90 (%)**	**KEGG (%)**	**Completeness (%)**
*A. monilatum*	68.0	79.7	73.2	99,594	31	38	17	83.47
*P. minimum*	70.0	42.7	58.3	96,684	23	29	14	76.61
*C. affinis*	11.8	20.0	14.1	19,072	40	51	27	89.11
*G. oceanica*	26.0	28.7	31.0	38,577	29	57	21	84.68
*C. polylepis*	45.3	23.2	29.0	53,786	39	41	18	81.45

*The combined assembly represents an assembly of all treatments. Annotations are the percent of combined assembly, which were annotated via the NCGR pipeline (listing the largest percent obtained from all four databases surveyed), uniref90 database, and KEGG*.

### Species-specific transcriptional patterns

**Figure 2:**
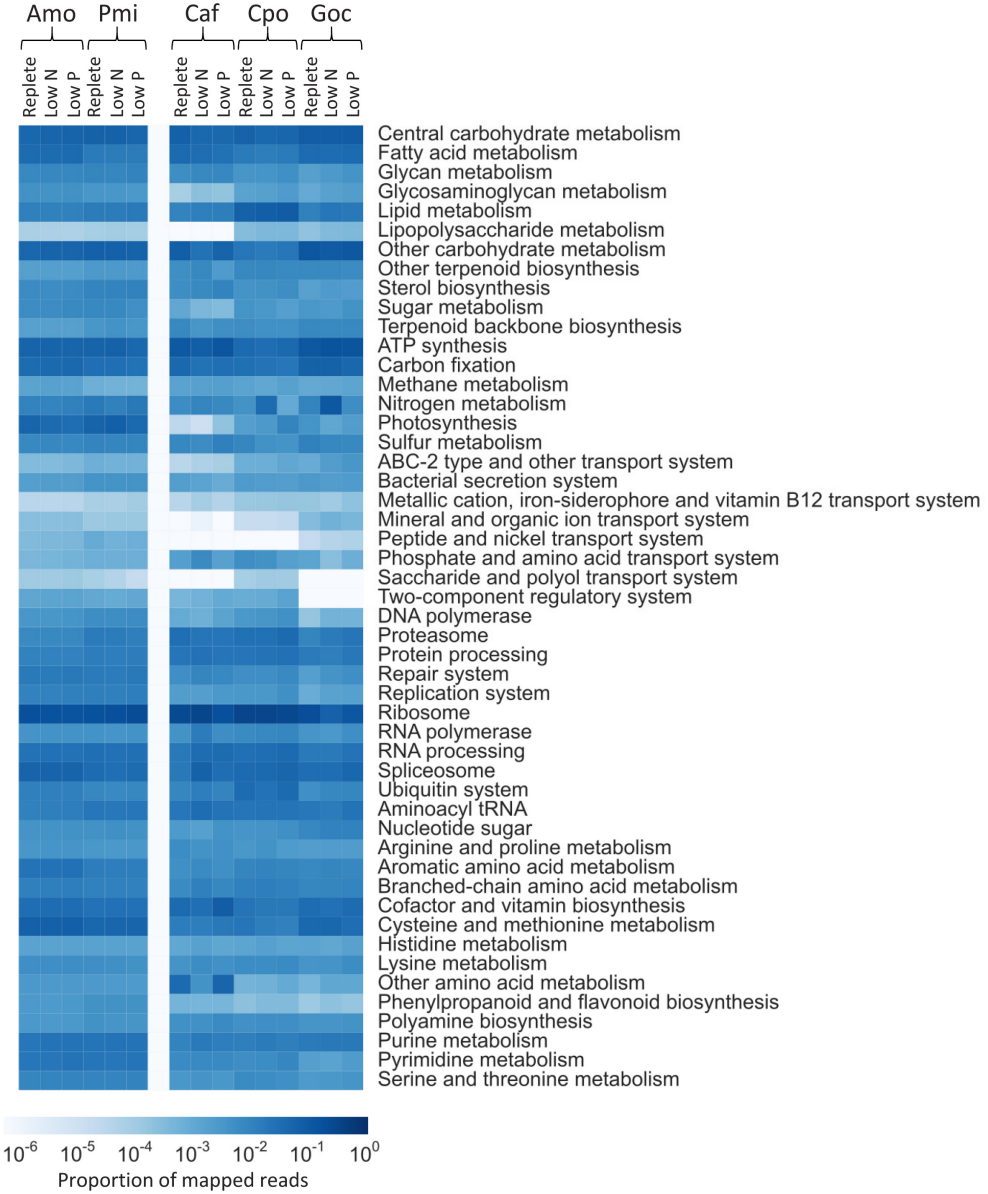
Quantitative metabolic fingerprint depicting the proportion of reads assigned to contigs with KEGG ID's and binned by KEGG module for each species and treatment. White denotes no reads were mapped. Species are abbreviated as follows: Amo, *A. monilatum*; Pmi, *P. minimum*; Caf, *C. affinis*; Cpo, *C. polylepis*; and Goc, *G. oceanica*.

The distribution of reads by KEGG modules highlighted differences in the metabolic response patterns between species, with clear differences in global transcriptional patterns between phylogenetic groups, and nutrient treatments within a group (Figure 2). Although using a KEGG framework represents only a subset of the data, it allows broad comparisons between species. The calcifying haptophyte *G. oceanica* had an increased proportion of reads mapping to central carbohydrate metabolism (9%), other carbohydrate metabolism (11%), ATP synthesis (14%), and carbon fixation (6%) compared to other species (Figure 2). Although the pathways for calcification are poorly understood, these differences likely in part underpin this species' unique biology. There was a notable increase in the proportion of reads mapping in the N metabolism module for the low N treatment, relative to the other treatments for *G. oceanica* (Figure 2). However, none of the aforementioned modules showed significant changes across treatments (Kolmogorov-Smirnov, *p* > 0.2, Figure S1). For the non-calcifying haptophyte *C. polylepis*, ribosomal and lipid metabolism pathways had a larger proportion of reads under all treatments (31 and 8%, respectively) vs. all other species (19 and 2% on average, respectively; Figure 2), perhaps reflective of this species' high lipid content (Bigelow et al., 2013). N metabolism was significantly modulated in *C. polylepis* (Kolmogorov-Smirnov, *p* = 0.05; Figure S2), concomitant with depressed photosynthetic efficiencies and high C:N ratios indicative of N stress (Table 1). The dinoflagellates were very similar to each other, but had a larger proportion of reads (0.07%) in the peptide and nickel transport system module relative to the other phyla (0.01%; Figure 2). Both of the dinoflagellates are known mixotrophs and the module read distribution may underpin this difference in trophic capability relative to the other phyla. The dinoflagellates displayed little variation between treatments or species, reflective of their limited transcriptional modulation (see below). The diatom *C. affinis* had an increased proportion of reads in the other amino acid metabolism module (4%), relative to other species (0.2%), and lacked reads mapping to the peptide and nickel transport system module among other variation (Figure 2). The genes involved in silica transport and metabolism are not represented in KEGG, but the differences observed here highlight variation in central metabolism for this diatom. Under low N, there was an decreased proportion of reads relative to the replete condition mapping to the cofactor and vitamin biosynthesis module (3 vs. 4%) and the ATP synthesis module (8 vs. 12%) while increases were observed in reads mapping to the spliceosome module (7 vs. 2%) and the ribosome module (ribosomal biogenesis proteins; 32 vs. 25%) (Figure 2). Significant differences were observed in both treatments relative to the replete for a number of these pathways (Kolmogorov-Smirnov, *p* < 0.01; Figure S3). These responses are consistent with the widespread transcriptional restructuring that happens in diatoms experiencing N and P stress (Bender et al., 2014), and the changes observed in physiology between treatments (Table 1). In summary, the KEGG-defined metabolic response patterns reflect the general transcriptional differences between phyla, and underpin the phenotypic changes associated with nutrient stress.

**Figure 3:**
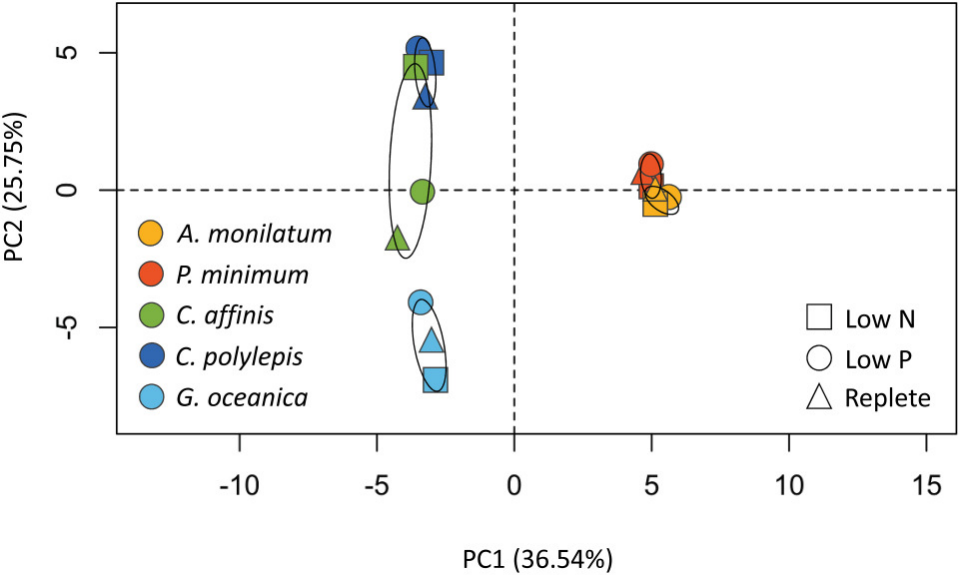
Principal component analysis (PCA) of the proportion of reads assigned to contigs with KEGG ID's and binned by KEGG module for each species and treatment. Confidence (95%) ellipses are depicted for each species. Species are coded by color and treatment by shape.

Principal component analysis (PCA) of the proportion of reads assigned to KEGG modules for each treatment, which were present in all species, was used to examine variation among species and treatments. This PCA analysis (Figure 3) identified variation between species (predominately PC1) and, to a lesser extent, treatments (predominately PC2). The two dinoflagellates were distinct from the other species in PC1, although each dinoflagellate, nevertheless, clustered with itself across treatments because of little variation in KEGG-defined metabolic responses (Figure 2). The haptophytes similarly clustered predominately by species rather than by treatment (Figure 3), although they displayed greater variation in KEGG-defined metabolic response between treatments compared to the dinoflagellates (Figure 2). The diatom displayed the greatest variation of KEGG-defined metabolic response between treatments as evidenced by the greater spread across PC2 (Figure 3). These patterns, generated on a subset of the data, are consistent with those observed in the whole dataset (Table 3). For example, the diatoms and haptophytes significantly modulated between 2 and 50% of their transcriptome (Table 3), consistent with what has been observed in other studies. In contrast, dinoflagellates significantly modulated only 0.1–1% of their transcriptomes (Table 3). The low levels of transcriptional changes observed in the two dinoflagellates in this study are consistent with prior research on dinoflagellates (Barshis et al., 2014; Murray et al., 2016). The majority of genes in dinoflagellates are post-transcriptionally regulated (Murray et al., 2016) with mRNA processed via spliced leader trans-splicing (Zhang et al., 2009). As such, the patterns observed here reflect consistent trends between species within a taxon and are congruent with the KEGG-defined metabolic responses (Figure 2), which mirrored the percentage of differentially abundant transcripts described above for each species (Table 3). In sum, these data highlight the species-specific differences that underpin the unique biology of these groups, and offer a rich database for future investigations.

**Table 3:** Significant differential abundance results with ASC (Wu et al., 2010) displaying the percentage of contigs and orthologs with significant increases (ITA) or decreases (DTA) in transcript abundance (post-*p* > 0.95 of fold change > 2) relative to the replete condition.

**Species**	**Treatment**	**Contigs**		**Orthologs**	
		**ITA (%)**	**DTA (%)**	**ITA (%)**	**DTA (%)**
*A. monilatum*	Low N	0.05	0.08	0.02	0.06
	Low P	0.13	0.13	0.01	0.07
*P. minimum*	Low N	0.04	0.09	0.00	0.02
	Low P	0.64	1.00	0.11	0.24
*C. affinis*	Low N	34.27	16.89	33.44	14.99
	Low P	19.66	9.84	13.79	10.63
*G. oceanica*	Low N	8.71	3.43	7.95	3.70
	Low P	8.64	2.07	8.29	2.11
*C. polylepis*	Low N	1.43	1.01	0.98	0.82
	Low P	3.86	3.16	3.42	2.92

### Conserved responses from orthologous group comparisons

**Figure 4:**
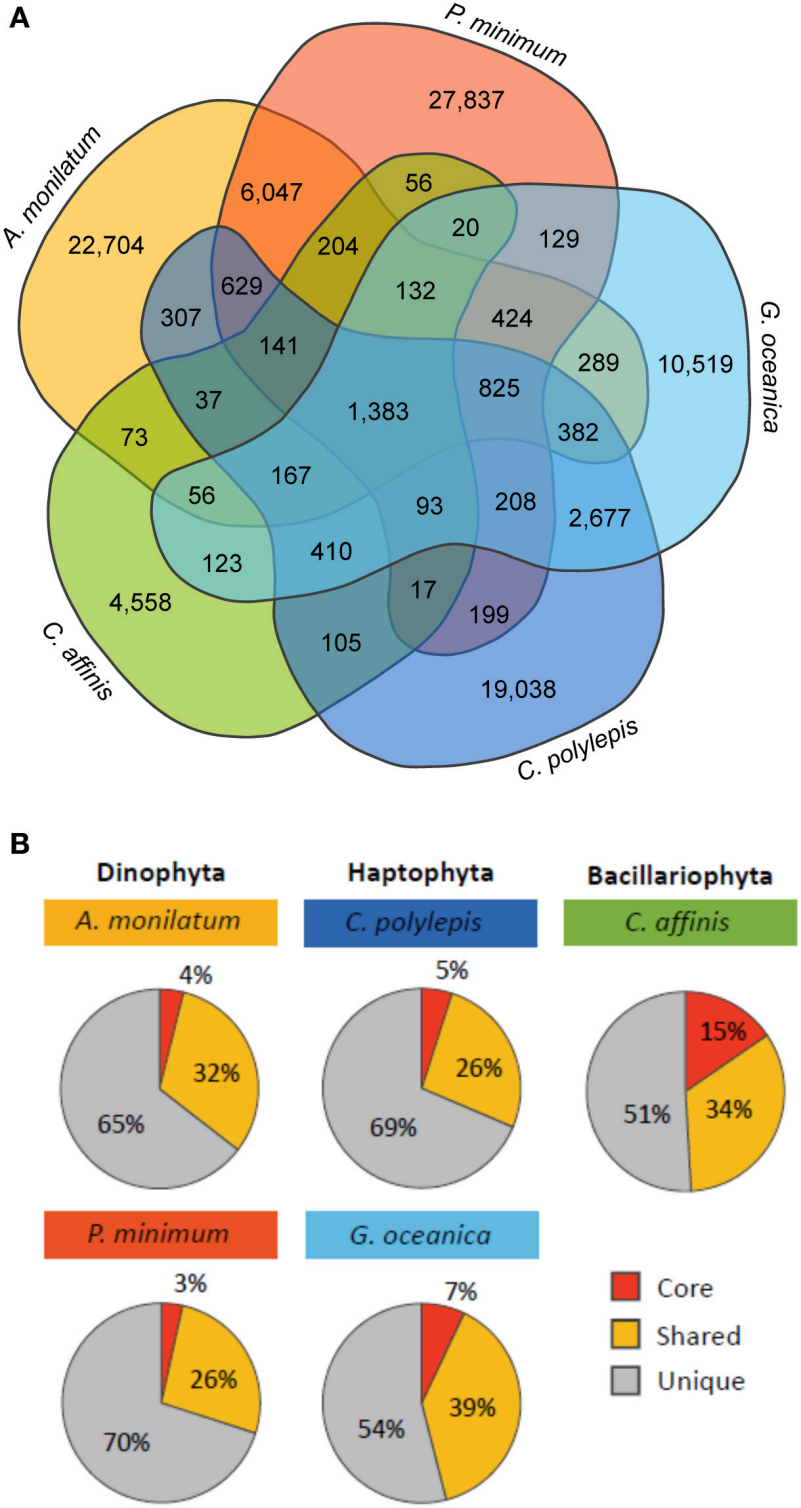
**(A)** Venn diagram displaying the number of orthologous groups shared between the five species and **(B)** the percent of total orthologous groups for each species that are core (shared by all species), shared (shared with one to three species), and unique (only found in that species).

Although species-specific differences in overall biochemical, metabolic, and transcriptomic response were observed, the primary goal of this study was to look for shared molecular responses in an effort to look for commonality. To investigate the relative conservation of molecular responses, an analysis of orthologous groups was conducted across the different taxa in this study. There were 1,383 orthologs common to each of the five species (Figure 4A) representing between 3 and 15% of orthologs for each species (core orthologs; Figure 4B). Annotations on this pool of core orthologs suggested many were related to essential metabolic pathways such as biosynthesis of amino acids and carbohydrate metabolism (Table S4), which is similar to other comparative analyses (Bender et al., 2014; Koid et al., 2014). Further, the contigs present in this core group mapped to 54–64% of CEGMA core eukaryotic genes and represented 70% on average of the core eukaryotic genes recovered by the whole transcriptome (Table S5). A PCA of these core orthologs grouped together taxa (e.g., haptophytes) and resolved minimal treatment variability, which is consistent with these core orthologs being related to essential metabolic pathways within phyla (Figure S4). Of the total orthologs within a particular species, 26 to 39% were shared with some subset of the other species (Figure 4B, “Shared”), with species of the same phyla sharing a greater percentage of orthologs with each other than with other organisms (Figure 4A). For example, the two haptophytes (*G. oceanica* and *C. polylepis*) shared 2,677 orthologs and the two dinoflagellates (*A. monilatum* and *P. minimum*) shared 6,047 orthologs while these two groups shared only 825 orthologs collectively (Figure 4A). The diatom *C. affinis* had the fewest shared orthologs as it formed its own taxonomic group, sharing between 56 and 123 orthologs with other species (Figure 4A). The majority of orthologs were unique to each species (51–70%; Figure 4B).

### Conserved responses to nutrient stress

**Figure 5:**
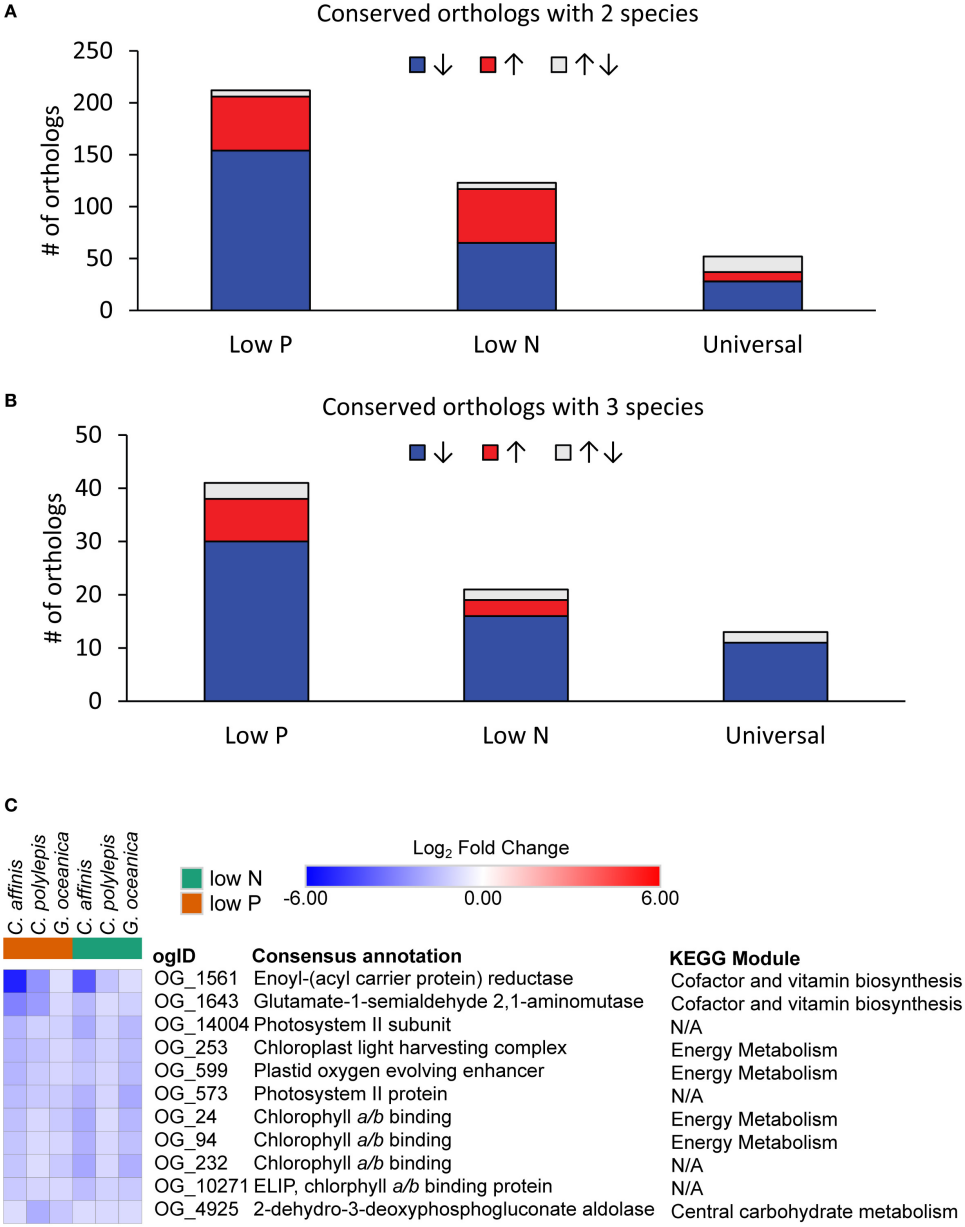
The distribution of conserved orthologous groups with significant (ASC, post-*p* > 0.95 of fold change > 2) differential abundance (log_2_-fold change relative to replete) where responses were shared between **(A)** two species and **(B)** three species for each treatment or in both treatments (Universal). Color denotes direction of abundance relative to replete with increased transcript abundance in red (↑), decreased transcript abundance in blue (↓), and both increased and decreased across species in gray (↑↓). **(C)** Heatmap of orthologs with universal response (all significantly decreased abundance across three species and both treatments). When no KEGG module could be identified, it was denoted as N/A.

Species-specific contributions (pooled contigs) to each orthologous group were analyzed for differential abundance in the low nutrient treatments relative to the replete to resolve differentially abundant orthologs that were conserved. The data had similar patterns across species to the patterns observed at the contig level described above (Table 3). Of the differentially abundant orthologs (27–3,645 depending on species and treatment; Table S2), 329 were identified as being conserved (Table S6), defined as orthologs that had significant increases or decreases in transcript abundance across at least 2 species in the same treatment (low N or low P) relative to the replete. The majority of conserved responses were shared between the haptophytes and diatom with only six conserved responses including a dinoflagellate (Table S6). The low representation of dinoflagellates in conserved orthologs was consistent with their low levels of differentially abundant orthologs and contigs. Further discussion of conserved orthologs will therefore focus only on the two haptophytes and diatom. Not surprisingly, more orthologs were shared between 2 compared to 3 species under both low N and low P (Figures 5A,B). Within these conserved orthologs, changes in relative abundance in response to either low N or low P overwhelming moved in the same direction across species, i.e., relative abundance generally either increased, or decreased, consistently in multiple species (Table S6). General differences in the patterns of shared orthologs between the low N and low P treatments were examined and a greater number of orthologs were conserved under only low P compared to the low N treatment (247 vs. 138, respectively; Figure 5, Table S6). Thus, the responses to N and P stress appear to vary in the degree to which molecular physiology is conserved across phytoplankton species, with greater conservation in the response to P stress. In a related transcriptomic study of the Raphidophyte, *Heterosigma akashiwo*, Haley et al. (in press) found greater metabolic diversity in the response to N compared to P stress. Similar observations have been reported for other phytoplankton and plants (Cai et al., 2011, 2012; Morey et al., 2011; Harke and Gobler, 2013). If the response to P stress potentially involves fewer metabolic pathways, there may be greater opportunity for responses to be conserved, supporting the observations herein.

The conserved orthologous group responses spanned a number of metabolic functions (for cases where the individual orthologs could be placed within metabolic pathways defined by KEGG) including carbon fixation, metabolism of N, sulfur, carbohydrate and lipid metabolism, nucleotide and amino acid metabolism, and biosynthesis of secondary metabolites (Table S6). Many of the conserved orthologs displayed decreased transcript abundance (between 53 and 85%; Figure 5), a reflection of the physiological responses to nutrient stress highlighted above (i.e., decreased growth rate, photosynthetic efficiency). For example, an ortholog identified as a chaperonin (OG2549), a protein involved in assembly and folding of other proteins (Levy-Rimler et al., 2002), had decreased transcript abundance in *C. affinis* and *G. oceancia* under N and P stress (Table S6). An ortholog identified as a peroxisome (OG10255) also decreased in abundance under N and P stress for *C. affinis* and *C. polylepis* (Table S6). Peroxisomes can have differing metabolic functions between species, but this response suggests a reduction in catabolic activity by nutrient-stressed cells (Gabaldon, 2010). Further, eleven orthologs displayed a universal nutrient response in the diatom and haptophytes (in that they were differentially abundant in both nutrient stresses for all three species; Figure 5B, “Universal”). Nine of these 11 universally responding orthologs were related to photosynthesis (Figure 5C), and all were significantly less abundant than the replete (ASC, post-*p* > 0.95 of fold change >2) mirroring the observed decrease in growth and photosynthetic efficiency in both low N and low P treatments (Table 1).

### Conserved nitrogen and phosphorus responses

In response to nutrient stress, phytoplankton employ a number of strategies to control intracellular nutrient levels for cellular survival including the transport of external sources of nutrients, the processing of organic matter and the recycling of internal stores (Roche et al., 1999; Moore et al., 2013; Dyhrman, 2016). Within the conserved orthologs, those specifically related to N and P metabolism were examined in detail to identify how they were modulated by N and P stress.

**Figure 6:**
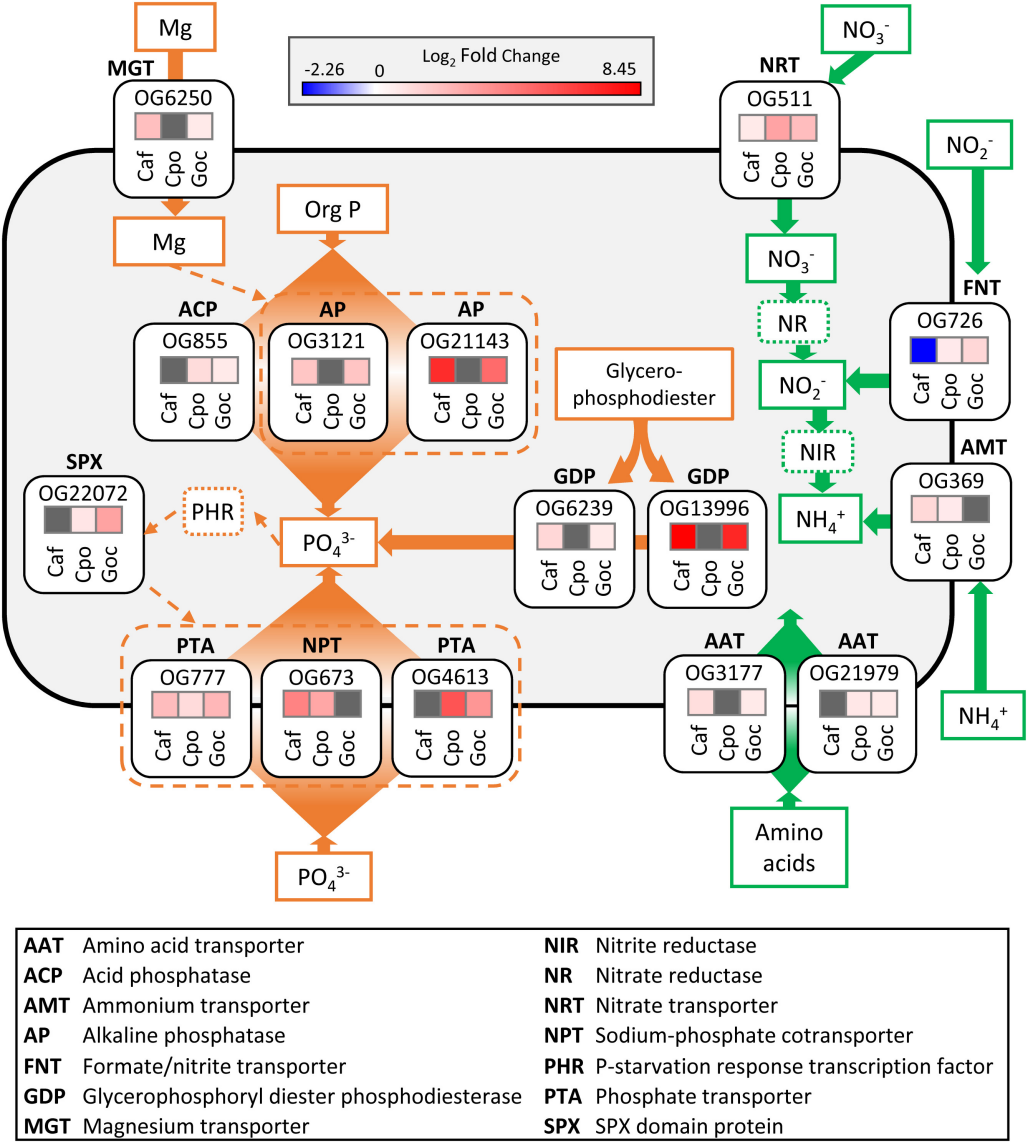
Schematic cell model depicting the significant (ASC, post-*p* > 0.95 of fold change > 2) ortholog responses associated with conserved nitrogen stress (green) and phosphorus stress (orange) responses in each species. Solid lines and arrows represent known pathways while dashed lines and arrows indicate potential pathways. For log_2_-fold change heat maps (treatment vs. replete), red colors denote increase in transcript abundance, blue colors denote decrease in transcript abundance, and gray denotes no differential expression. Orthologous group ID is above each fold change heat map box. The dinoflagellates were not included in this analysis. Caf, *C. affinis*; Cpo, *C. polylepis*; Goc, *G. oceanica*.

The majority of the world's oceans and coastal regions are characterized by N limitation (Dugdale, 1967; Moore et al., 2004) and the rate of N supply to these waters is considered the principal driver of primary productivity in a range of different systems (Vitousek and Howarth, 1991; Zehr and Kudela, 2011). N stress is known to elicit strong influence on gene expression profiles across a range of different phytoplankton taxa (Tolonen et al., 2006; Bender et al., 2014; Frischkorn et al., 2014; Levitan et al., 2015). This response is typically characterized, in part, by the modulation of transporters for different N forms (Raven and Giordano, 2016). Here, there was significantly increased abundance across two or more species for orthologous groups corresponding to a nitrate transporter (OG511), a formate/nitrite transporter (OG726), amino acid transporters (OG3177 and OG21979), and an ammonium transporter (OG369) (Figure 6).

A nitrate transporter ortholog (OG511) increased in abundance during N stress for *C. affinis, C. polylepis*, and *G. oceanica* (Figure 6), representing a response conserved across all three species (Figure 6, Table S6). Consistent with these results, nitrate transporters increased with N stress in a number of diatom genera (Bender et al., 2014; Kang et al., 2015). In the haptophyte, *E. huxleyi*, a nitrate transporter (*Nrt2*) was up-regulated 2-fold under N starvation (Song and Ward, 2007), and similar responses were observed in the haptophytes, *Isochrysis galbana* and *Prymnesium parvum* (Kang et al., 2007; Liu et al., 2015). Nitrate transporters therefore appear to be important markers for cellular N demand in diatoms and haptophytes, and markers that are widely shared, such as this ortholog, may be particularly useful in that context.

In addition to the shared nitrate transporter response, an ortholog (OG726) identified as a formate/nitrite transporter displayed increased transcript abundance in the N stressed haptophytes (*C. polylepis* and *G. oceanica*) but decreased transcript abundance in the diatom *C. affinis* (Figure 6). Transcription of this family of transporters has been found to increase under P stress in the haptophyte, *P. parvum* (Beszteri et al., 2012), decrease when grown on urea vs. nitrate in cells of an unidentified cryptophyte (Terrado et al., 2015), and increase under N limitation for the pelagophyte, *Aureococcus anophagefferens* (Berg et al., 2008). This variable response across taxa is likely reflective of available nitrite levels within the cell rather than external concentrations (Galván, 2002; Maeda et al., 2014).

An ammonium transporter ortholog (OG369) increased in abundance under N stress in both *C. affinis* and *C. polylepis* (Figure 6). Many eukaryotic phytoplankton appear to have multiple copies of ammonium transporters (AMTs) with varying transcriptional response to N stress. For example, in the diatoms *Cylindrotheca fusiformis* and *T. pseudonana* some AMTs are highly expressed under N starvation while others were not (Armbrust et al., 2004; Hildebrand, 2005; Bender et al., 2014). Further, ammonium appears to be an important nutrient for *E. huxleyi* as its pan-genome contains 20 AMTs (Read et al., 2013). The diversity of AMTs and differences in their expression patterns are perhaps indicative of multi-phasic uptake systems observed in higher plants and some phytoplankton (Joseph et al., 1975; Dogar and van Hai, 1977; Ower and Cresswell, 1986). Although there is much diversity among phytoplankton AMTs, orthologous genes often retain the same function over the course of evolution (Li et al., 2005), as evidenced by the current finding.

Two amino acid transporter orthologous groups (OG3177 and OG21979) had increased transcript abundance relative to the replete in both the diatom and haptophytes (Figure 6). In N-limited regions of the ocean, dissolved organic N concentrations are often high relative to dissolved inorganic N and may represent an important alternative source of N to phytoplankton (Bronk et al., 2007). Some phytoplankton have been shown to grow on amino acids as a sole N source (Baden and Mende, 1979; Ietswaart et al., 1994; John and Flynn, 1999; Mary et al., 2008) and direct uptake using ^14^C-labeled amino acids has been demonstrated in 25 marine phytoplankton including diatoms, haptophytes, and green algae (Wheeler et al., 1974). Transcription of amino acid transporters in response to N stress has been observed in pelagophytes (Wurch et al., 2011b; Frischkorn et al., 2014), diatoms (Hockin et al., 2012; Bender et al., 2014; Alexander et al., 2015a), and marine and freshwater prokaryotes (Gifford et al., 2013; Harke and Gobler, 2015). Nevertheless, although the change in abundance of these two amino acid transporters is consistent with prior studies, only these two orthologs were conserved.

Collectively, the N transporters conserved in the diatom and haptophytes tended to increase in abundance in response to N stress, mirroring observations in previous studies of other diatoms and haptophytes, as well as studies of other phytoplankton such as pelagophytes (Berg et al., 2008; Frischkorn et al., 2014; Dupont et al., 2015), prasinophytes (McDonald et al., 2010) and marine cyanobacteria (Tolonen et al., 2006). However, very few orthologous groups for N transport functions were shared among even two species. This highlights the diversity of molecular mechanisms used by different species, even related species, in response to similar environmental stress. Because they are so few, the conserved N transporters in this study are also highlighted as particularly interesting for further research.

Compared to conserved N responses, there were more conserved ortholog responses to P stress (Figure 5, Table S6). These conserved responses were primarily driven by the decreased abundance in orthologs associated with protein, amino acid, and ribosomal protein synthesis, and increased abundance in orthologs associated with the assimilation of inorganic P and acquisition of P from organic substrates (Table S6). Conserved orthologs with functions related to P acquisition and transport were examined in greater detail. It is increasingly recognized that the P stress response in phytoplankton involves increased hydrolysis of organic phosphorus, either to modulate intracellular stores or to grow on exogenous dissolved organic phosphorus (DOP; Dyhrman and Ruttenberg, 2006; Dyhrman et al., 2007; Orchard et al., 2009; Martin et al., 2014). In some cases, these shifts appear to be controlled at the transcriptional-level (Dyhrman et al., 2012). Here, two orthologs defined as glycerophosphoryl diester phosphodiesterase (GDP; OG6239 and OG13996) had increased transcript abundance in both *C. affinis* and *G. oceanica* under P stress (Figure 6). A number of phytoplankton can grow on phosphodiesters as a sole P source (Oh et al., 2002; Moore et al., 2005; Yamaguchi et al., 2013), and the GDP enzyme may act on exogenous DOP. In addition, this enzyme can hydrolyze diesters like phospholipids, so it may also be involved in the turnover of phospholipids that occurs in phytoplankton including both diatoms and haptophytes (Van Mooy et al., 2009; Martin et al., 2011). GDP has previously been described as significant in the P stress response of *T. pseudonana* (Dyhrman et al., 2012) and field populations of *Skeletonema* spp. (Alexander et al., 2015a). Significant reduction of transcripts with P addition for this enzyme have also been detected in diatom populations (relative to a control) present in the North Pacific subtropical gyre (NPSG) (Alexander et al., 2015b). The presence of these transcripts and their consistent response to P stress in laboratory and field populations of diatoms provides strong support that GDP is a highly conserved P stress response in the diatoms. The conservation of this response in *G. oceanica* suggests that this enzyme can also be an important aspect of the P stress response in other taxa. However, the lack of conserved response in *C. polylepis* (despite detection of its presence; Table S6) indicates variability within haptophytes. Notably, haptophyte GDP transcripts were detected in the NPSG, but were not significantly decreased by P addition (Alexander et al., 2015b). This is consistent with the variability in GDP response observed between haptophytes herein.

Alkaline phosphatase (APase) enzymes also mediate DOP hydrolysis in many phytoplankton (Dyhrman, 2016), and they are typically highly expressed in low P conditions to facilitate a switch to growth on phosphomonoesters (Cembella et al., 1984). Two APase orthologs (OG3121 and OG21143) had a conserved response, with increased abundance in the low P treatment relative to the control in *C. affinis* and *G. oceanica* (Figure 5). Concomitant with the change in relative transcript abundance, APase activity was significantly increased in the low P treatment relative to the control for *G. oceanica* (Table S3). P-dependent induction of an APase transcript was observed in *C. affinis* strain CCMP 160 (Shih et al., 2015), consistent with the observation here and parallels to *G. oceanica* can be inferred from transcription of APases in *E. huxleyi* (Xu et al., 2006, 2010). It is increasingly recognized that APase activity is a common response to P stress in phytoplankton (Dyhrman, 2016).

Additionally, there was one conserved response in an acid phosphatase (APC; OG855), which had significantly increased abundance in the low P treatment relative to the control for the haptophytes *C. polylepis* and *G. oceanica* (Figure 5). Like alkaline phosphatases, acid phosphatases have broad specificity against different substrates but are optimally active at pH lower than 7 and seem to be involved in internal cell metabolism (Jansson et al., 1988). Acid phosphatases have been shown to increase in activity under low P conditions in *Euglena gracilis* (Blum, 1965) and *C. affinis* (Møller et al., 1975). It is likely that these enzymes, in the current study are actively recycling organic substrates in the face of P stress and may represent an additional strategy for haptophytes living in low P environments.

APase enzymes contain metal cofactors that constitute active sites with a direct role in catalysis (Stec et al., 2000). For example, the APase characterized in *E. coli* has both magnesium and zinc as cofactors (Bradshaw et al., 1981). Although the cofactors required by eukaryote APases, which include calcium and zinc, are not well defined in most cases (Dyhrman, 2016), there was a conserved increase in the abundance of a magnesium transporter (OG6250) in both *C. affinis* and *G. oceanica* under P stress (Figure 5). More research is warranted to identify if this is related to the increase in APA for these species or if other biological processes are involved.

Maintenance of phosphate homeostasis in higher plants and yeast is thought to be controlled by processes involving the SPX domain (Secco et al., 2012). SPX domain proteins accumulate during P stress and are reported to play an important role in the phosphate sensing and response system (Lv et al., 2014; Zhou et al., 2015), indirectly regulating aspects of the P stress response. SPX domain transcripts have been found to be significantly more abundant in P stressed cells relative to controls in the pelagophyte, *A. anophagefferens* (Frischkorn et al., 2014). Here, numerous orthologs, with SPX domain annotations and spanning all three phyla, were identified (Table S6). However, only one ortholog (OG22072) encoding a SPX domain protein displayed a conserved response with increased transcript abundance in both haptophytes (Figure 5). Detection and function of the SPX domain in eukaryotic phytoplankton remains largely uncharacterized (Lin et al., 2016) and additional studies are needed to examine the distribution and regulation of SPX domain genes in other genera of phytoplankton and its role in the phytoplankton P stress response.

Eukaryotic phytoplankton typically have multiple copies of phosphorus transporters and a common response to P stress is to increase transcription of P transporters (Dyhrman, 2016). For instance, the diatom *T. pseudonana* increased both transcription and protein abundance of a P transporter under P stress (Dyhrman et al., 2012) and, similarly, *E. huxleyi* increased transcription of a P transporter and a P repressible permease (Dyhrman et al., 2006b; McKew et al., 2015). Similar responses have been observed in *Tetraselmis chui* (Chung et al., 2003), *A. anophagefferens* (Wurch et al., 2011a), and *Chlamydomonas reinhardtii* (Moseley et al., 2006). In the current study, three phosphate transport orthologs (OG777, OG673, and OG4613) were identified with conserved responses in the diatom and haptophytes, and all had significantly increased abundance under P stress. Although P transporters were detected in the dinoflagellate transcriptomes examined herein (Table S4), significant changes in the relative abundance of these orthologs were not observed with P or N stress (Table S2). This lack of transcriptional response has been attributed to a general lack of transcriptional level regulation in the dinoflagellates (Morey et al., 2011), which could be resolved with future proteomic studies for the dinoflagellates.

## Conclusion

In the current study, broad, species-specific physiological and transcriptomic responses were observed highlighting differing metabolisms that underpin the unique biology of these groups. At the ortholog level, most differentially-abundant responses were also species-specific, which underscores the diversity of molecular mechanisms employed by these phytoplankton in response to N and P stress. However, the goal of this study was to identify molecular response commonalities and a number of responses conserved across taxonomic groups were found in the diatom and haptophytes. Some of these conserved responses were related to observed phenotypic changes in biogeochemical pools and activities. For example, many of the orthologs that were conserved under both N and P stress were related to photosynthesis. These orthologs likely represent generalized stress responses that likely extend beyond the phytoplankton groups studied here. More conserved responses were found for P stress compared to N stress, suggesting that eukaryotic phytoplankton may have less flexibility in how they alter metabolic processes at the molecular level in response to P stress. Orthologs that were identified as being conserved only in response to either N or P stress, in particular those that likely have roles in N and P transport and metabolism, may be targets for identifying the physiological status of field populations. Ultimately, identifying taxon-level differences, and similarities, in N and P stress responses contributes to our understanding of how changing nutrient environments can modulate phytoplankton community dynamics.

## Author contributions

MH: Data analysis and writing. AJ: Experimental concept/design, experiment execution, data analysis, and writing. SH: Experiment execution, sample processing, data analysis, and writing. HA: Data analysis and writing. SD: Experimental concept/design and writing.

### Conflict of interest statement

The authors declare that the research was conducted in the absence of any commercial or financial relationships that could be construed as a potential conflict of interest.
