# Supplementary material for: Conserved Transcriptional Responses to Nutrient Stress in Bloom-Forming Algae
Source: Front Microbiol. 2017 Jul 18;8:1279. doi: 10.3389/fmicb.2017.01279 (PMC5513979; doi:10.3389/fmicb.2017.01279)

Supplementary Material

**Conserved transcriptional responses to nutrient stress in bloom-forming algae.**

**Matthew J. Harke, Andrew R. Juhl, Sheean T. Haley, Harriet Alexander, Sonya T. Dyhrman^*^**

*** Correspondence:** Sonya T. Dyhrman, sdyhrman@ldeo.columbia.edu

# Supplementary Data

Tables S1, S2, S4, and S6 are located in separate files as they are too large to include in this supplementary material file. The Table legends are included below.

**Table S1** Differential expression results (ASC, post-*p* > 0.95 of fold change >2) for contigs of each species for treatments relative to the control. Species abbreviations are as follows: amo = *A. monilatum*, pmi = *P. minimum*, caf = *C. affinis*, cpo = *C. polylepis*, and goc = *G. oceanica*.

**Table S2** Differential expression results (ASC, post-*p* > 0.95 of fold change >2) for orthologs of each species for treatments relative to the control. Species abbreviations are as follows: amo = *A. monilatum*, pmi = *P. minimum*, caf = *C. affinis*, cpo = *C. polylepis*, and goc = *G. oceanica*.

**Table S4** Complete list of assigned ortholog ID, contig ID, annotations, and non-normalized read counts for all species in the study. Where no annotation was retrieved, an "n/a" is designated. Contig ID's are formatted with species abbreviation and contig number from NCGR assemblies. Species abbreviations are as follows: amo = *A. monilatum*, pmi = *P. minimum*, caf = *C. affinis*, cpo = *C. polylepis*, and goc = *G. oceanica*.

**Table S6** Conserved orthologous groups with significant (ASC, post-*p* > 0.95 of fold change >2) differential abundance (log2 fold change) of low P and low N relative to the replete.

# Supplementary Tables and Figures

**Table S3** Alkaline phosphatase activity (nmol P h^-1^ cell^-1^) for replicate experiments conducted after sequencing. Values represent the mean (n = 3) with coefficient of variation (CV), the ratio of standard deviation to the mean, in parentheses. Asterisks indicate significant differences from replete, where on asterisk is p < 0.05, two asterisks are p < 0.005 and three asterisks are p < 0.0005 (unpaired t-test).

|  | Replete | Low P |
| --- | --- | --- |
| *C. polylepis* | 6.3E-05 (53%) | 4.4E-03 (11%)*** |
| *G. oceanica* | 3.2E-04 (12%) | 2.9E-02 (25%)** |
| *P. minimum* | 5.7E-04 (11%) | 9.1E-03 (39%)* |

**Table S5** Cegma completeness comparison between completeness scores for each transcriptome (Total), core orthologous group (core), and percent of total core eukaryotic genes recovered by the whole transcriptome.

| **Species** | **Total** | **Core** | **% Core of Total** |
| --- | --- | --- | --- |
|  | **Completeness** | **Completeness** | **Completeness** |
| *A. monilatum* | 83.47% | 59.68% | 71.50% |
| *P. minimum* | 76.61% | 54.44% | 71.06% |
| *C. affinis* | 89.11% | 63.71% | 71.50% |
| *G. oceanica* | 84.68% | 59.68% | 70.48% |
| *C. polylepis* | 81.45% | 54.44% | 66.84% |

Nitrogen metabolism


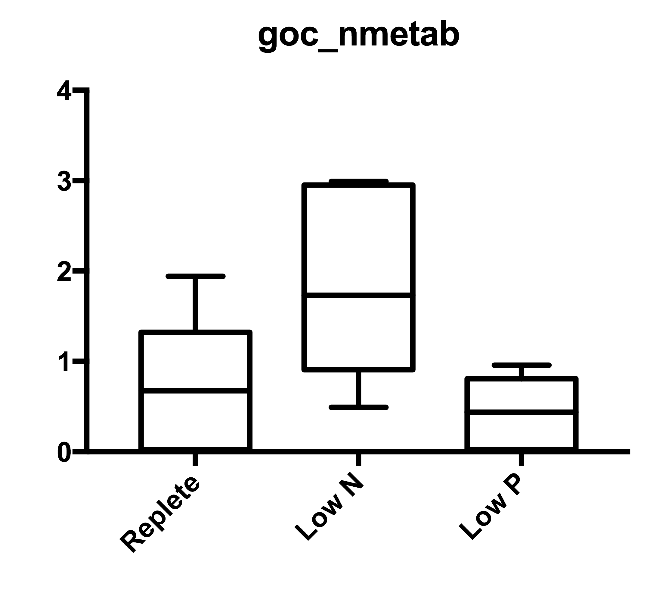


**A**

Distribution of

normalized enrichment


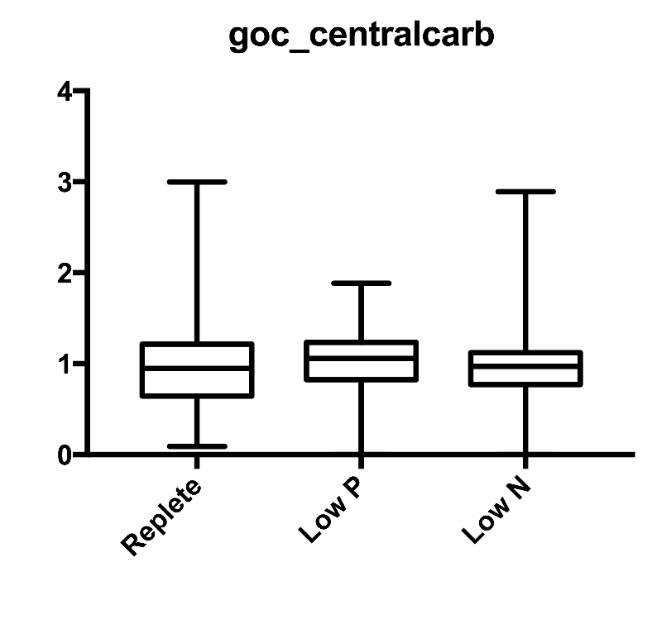


**B**

Central Carbohydrate Metabolism


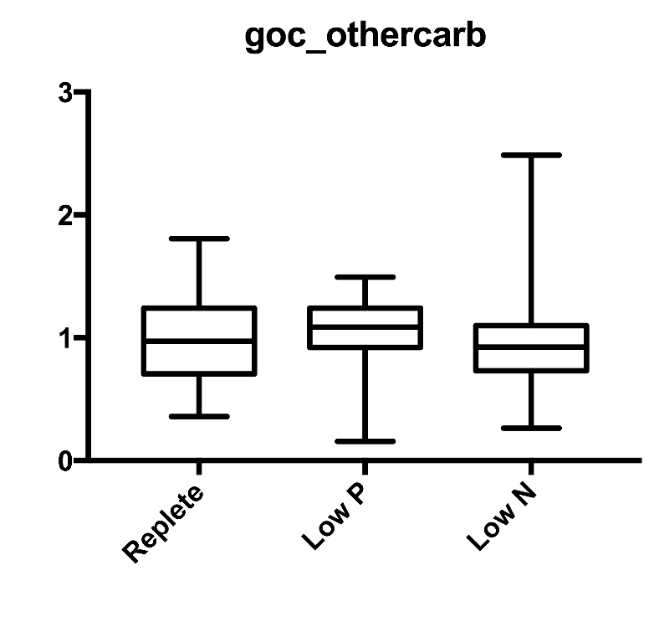


Distribution of

normalized enrichment

Other Carbohydrate Metabolism

**C**

**Figure S1** Box and whisker plots of KEGG pathway enrichment patterns for *G. oceanica*. Each plot represents the variation in expression (TPM) between treatments for contigs with KEGG IDs involved in each pathway noted above the plot. No significant difference was observed between treatments relative to the replete (Kolmogorov-Smirnov, *p* > 0.05).


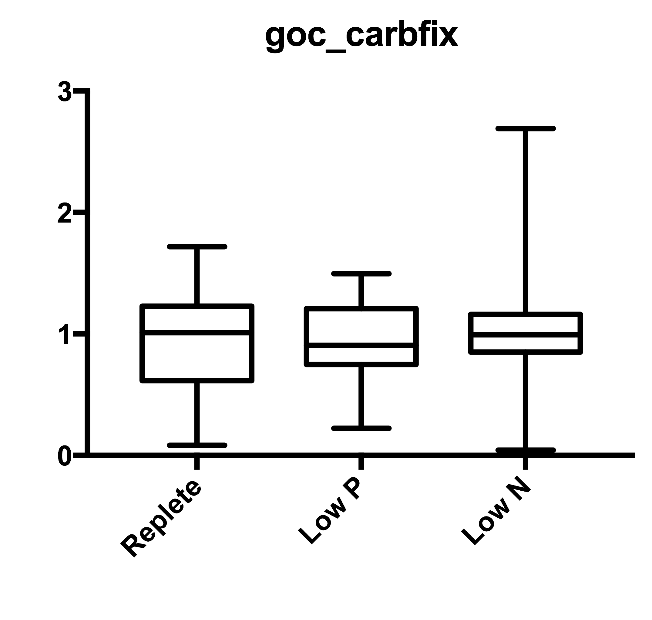


Carbon Fixation

**D**

**Figure S2** Box and whisker plots of KEGG pathway enrichment patterns for *C. polylepis*. Each plot represents the variation in expression (TPM) between treatments for contigs with KEGG IDs involved in each pathway noted above the plot. A Komorogov-Smirnov test was conducted to infer significant differences from the replete condition at *p* ≤ 0.05, denoted with a star.

Nitrogen metabolism

**B**

Distribution of

normalized enrichment

Lipid & Lipopolysaccharide metabolism


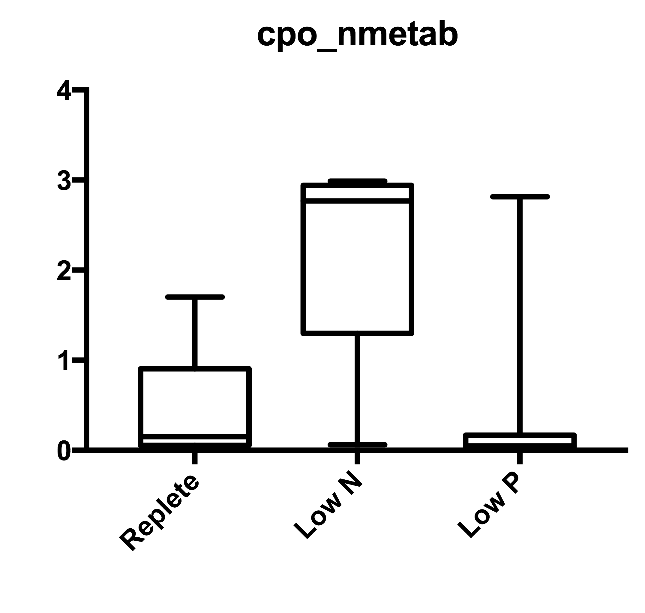


**A**

Distribution of

normalized enrichment


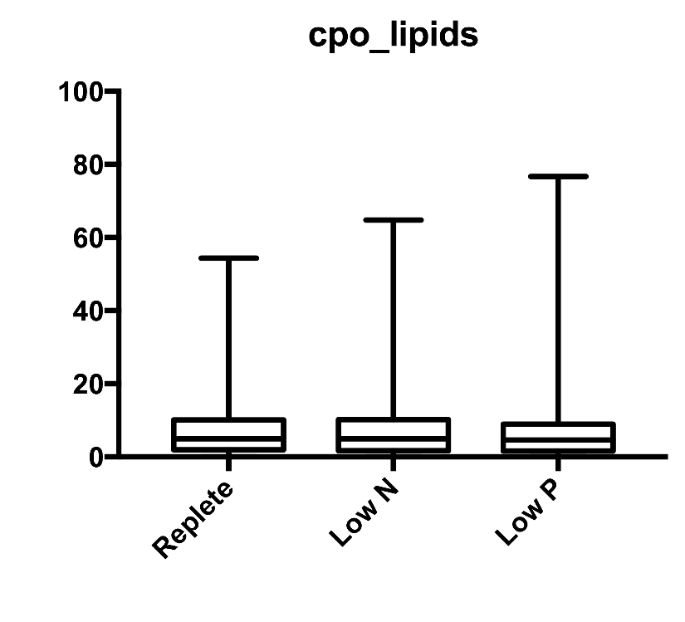


**Figure S3** Box and whisker plots of KEGG pathway enrichment patterns for *C. affinis*. Each plot represents the variation in expression (TPM) between treatments for contigs with KEGG IDs involved in each pathway noted above the plot. A Komorogov-Smirnov test was conducted to infer significant differences from the replete condition at p ≤ 0.05, denoted with a star.


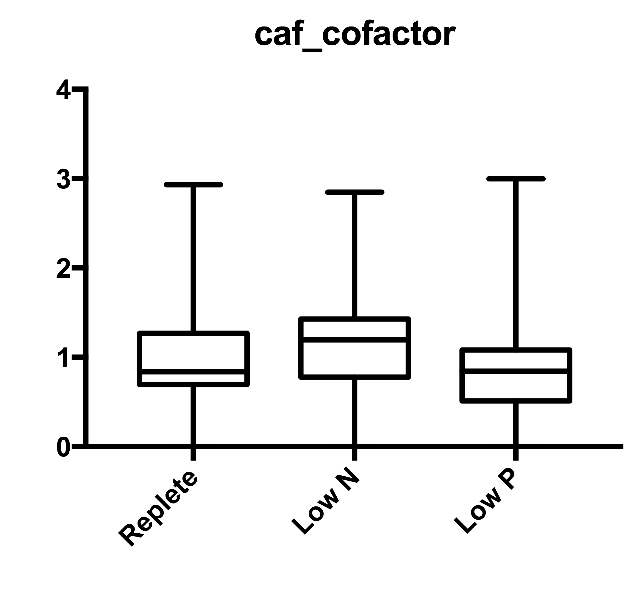

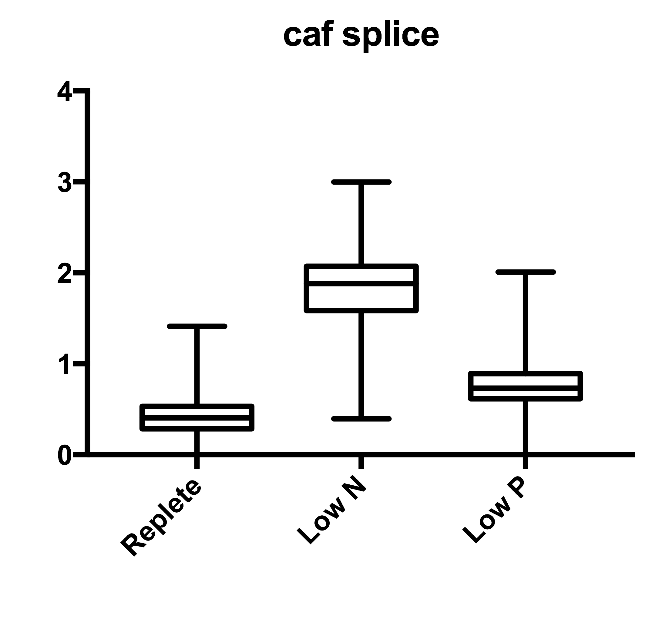


Spliceosome


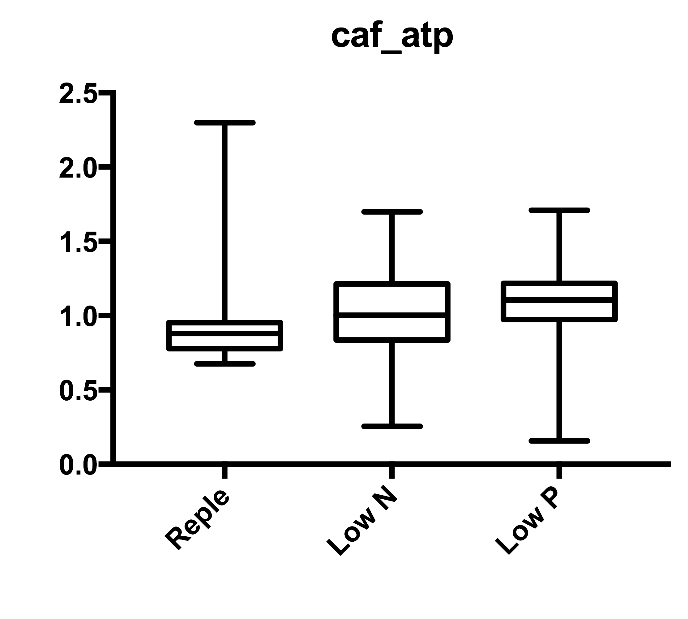

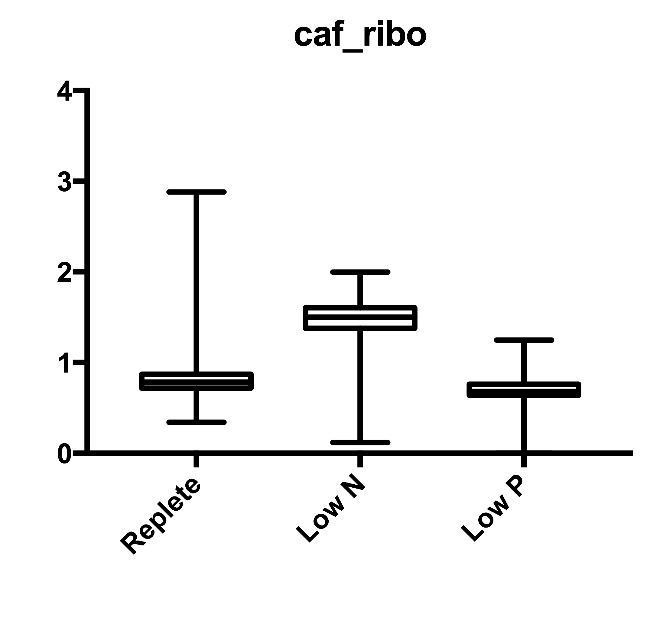


ATP Synthesis

Cofactor and vitamin biosynthesis

Ribosome

Distribution of

normalized enrichment

Distribution of

normalized enrichment

Distribution of

normalized enrichment

Distribution of

normalized enrichment

**Figure S4** Principal component analysis (PCA) of the normalized read counts assigned to the core orthologous group (shared orthologs). Species are coded by color and treatment by shape.


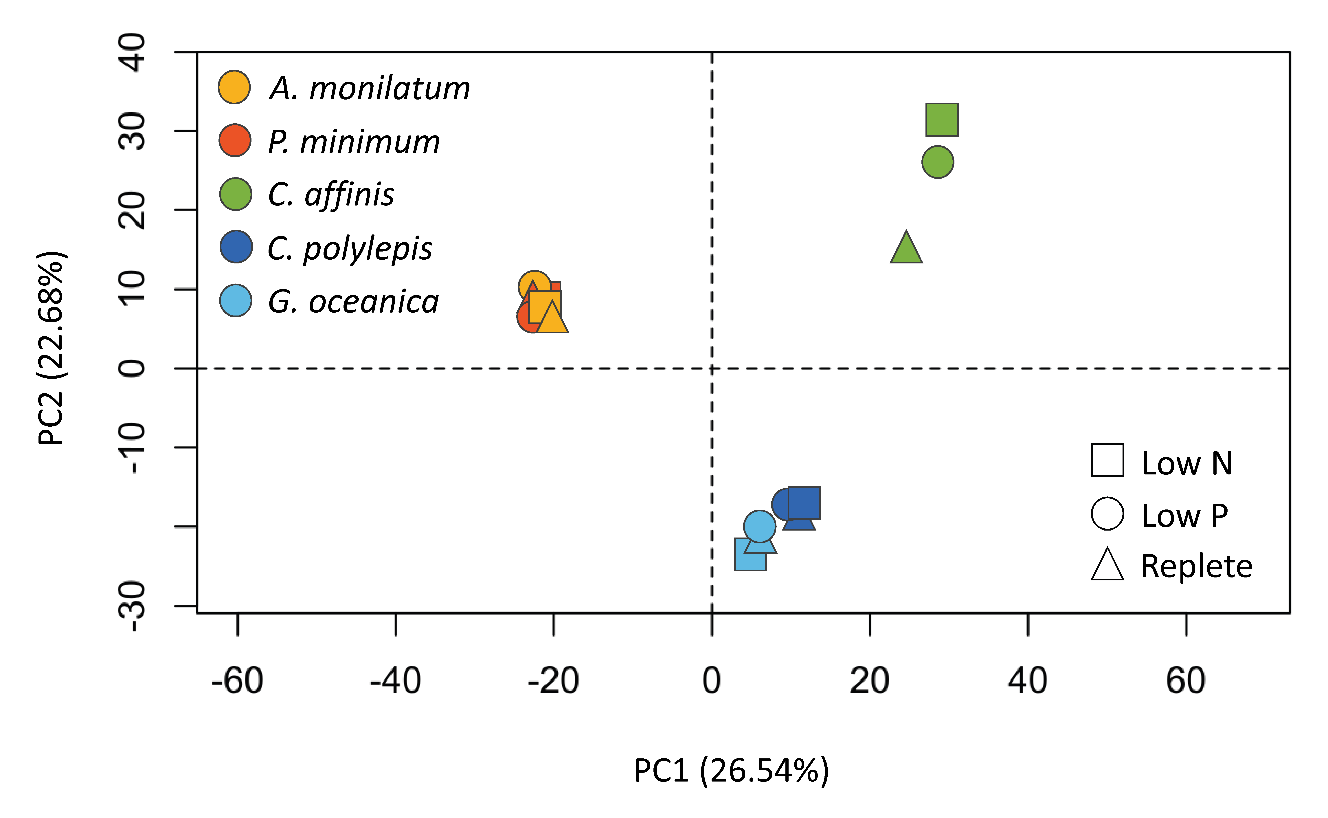

Supplement: Supplementary file 5 [file DataSheet1.DOCX]
